# Application and Research Progress of Covalent Organic Frameworks for Solid-State Electrolytes in Lithium Metal Batteries

**DOI:** 10.3390/ma16062240

**Published:** 2023-03-10

**Authors:** Yufeng Qiao, Xiaoyue Zeng, Haihong Wang, Jianlin Long, Yanhong Tian, Jinle Lan, Yunhua Yu, Xiaoping Yang

**Affiliations:** 1 State Key Laboratory of Organic-Inorganic Composites, College of Materials Science and Engineering, Beijing University of Chemical Technology, Beijing 100029, China; 2 Key Laboratory of Carbon Fiber and Functional Polymers, Ministry of Education, Beijing University of Chemical Technology, Beijing 100029, China

**Keywords:** covalent organic frameworks (COFs), solid-state electrolyte, lithium metal batteries, ion conductors

## Abstract

Covalent organic frameworks (COFs) are a class of crystalline porous organic polymers with periodic networks that are constructed from small molecular units via covalent bonds, which have low densities, high porosity, large specific surface area, and ease of functionalization. The one-dimension nanochannels in COFs offer an effective means of transporting lithium ions while maintaining a stable structure over a wide range of temperatures. As a new category of ionic conductors, COFs exhibit unparalleled application potential in solid-state electrolytes. Here, we provide a comprehensive summary of recent applications and research progress for COFs in solid-state electrolytes of lithium metal batteries and discuss the possible development directions in the future. This review is expected to provide theoretical guidance for the design of high-performance solid-state electrolytes.

## 1. Introduction

New energy resources are receiving increasing attention to avoid environmental pollution, global warming, and the greenhouse effect, which can be derived from the overuse of fossil fuels. Because the supply of renewables such as wind and solar energy is intermittent, which underscores the urgent need for efficient energy storage systems. Secondary battery technologies offer a promising pathway for sustainable energy supply [1,2,3]. The lithium-ion batteries (LIB) have emerged as a leading candidate due to their high energy density, wide electrochemical stability window, and long cycle life, making them suitable for use in portable electronic devices and electric vehicles [4,5]. Considering the limited theoretical capacity of commercialized graphite anodes (372 mAh g^−1^), Li metal anodes can better satisfy the desire for enhanced energy density due to ultrahigh theoretical specific capacity (3860 mAh g^−1^), low redox potential (−3.040 V versus standard hydrogen electrode) and low density (0.56 g cm^−3^ at room temperature) [6,7]. However, Li metal anodes can lead to side reactions with organic liquid electrolytes, and the uncontrolled growth of lithium dendrites and the formation of dead lithium pose severe safety hazards of Li metal batteries (LMB). Correspondingly, compared with liquid electrolytes, solid-state electrolytes (SSEs) offer excellent mechanical properties and are expected to suppress the dendritic Li metal formation, leading to safe operation of high-capacity batteries. For this reason, SSEs have gradually become a research hotspot [8,9].

Typically, the widely investigated SSEs can be classified into three categories: solid polymer electrolytes (SPEs), solid inorganic electrolytes (SIEs), and composite solid-state electrolytes (CSSEs). SPEs are usually based on a polymer matrix such as poly (ethylene oxide) (PEO), poly (vinylidene fluoride) (PVDF), polyacrylonitrile (PAN), and poly (methyl methacrylate) (PMMA). On the one hand, SPEs have high flexibility, low flammability, lightweight, good processability, and intimate interfacial contact with electrodes. On the other hand, SPEs have the drawback of limited ionic conductivity at room temperature, which restricts their practical application [10,11]. SIEs are mainly oxides and sulfides, such as garnet Li_7_La_3_Zr_2_O_12_ (LLZO), perovskite Li_3.3_La_0.56_TiO_3_ (LLTO), superionic sodium conductors (NASICON), Li_10_GeP_2_S_12_ (LGPS), which exhibit excellent ionic conductivity and rigidity. However, they also have some drawbacks, including brittleness, poor processability, and interfacial stability when compared with SPEs [12,13]. Past research suggested that the integration of inorganic fillers into a polymer matrix can enhance the ionic conductivity of the electrolytes while maintaining the characteristics of both rigidity and flexibility. Therefore, CSSEs have made significant progress by integrating the advantages of SPEs and SIEs [14,15,16]. Even so, there are still challenges, such as undesirable room temperature conductivity [17,18], low lithium-ion transference numbers, and poor interfacial compatibility in CSSEs. Therefore, it is necessary to develop advanced solid-state electrolytes to meet the needs of future batteries.

Covalent Organic Frameworks (COFs) are a class of crystalline porous organic polymers with periodic lattices and ordered nanopores. COFs are composed of light elements from the first and second periods, such as C, H, O, N, and B, by strong covalent bonds. COFs exhibit low density (as low as 0.17 g cm^−3^), tunable pore sizes (0.52–6.5 nm), and large specific surface area (ranging from a few hundred to a few thousand m^2^ g^−1^). According to the building block dimension, COFs can be divided into two-dimensional (2D) and three-dimensional (3D) ones [19,20,21,22]. In 2005, Yaghi and his co-workers were the first to synthesize COFs, namely COF-1 and COF-5, through reversible covalent bonds [23]. These materials have received widespread attention due to their tailorable structures and functional groups, which can make them versatile in applications such as gas separation [24], drug delivery [25], catalysis [26,27], energy storage [28], and proton conduction [29]. Compared with the earlier classical porous materials, Metal-Organic Frameworks (MOFs), COFs are more chemically stable on account of their unique covalent linkages [30,31,32]. Accordingly, COFs have more potential for electrochemical applications [33]. COFs are able to maintain stable, permanent porosity and polycrystalline structure over a broad temperature range, thus avoiding the insufficient room-temperature ionic conductivity of the conventional linear polymer. Additionally, the ordered one-dimensional (1D) nanochannels of 2D COFs provide sufficient free volume to steadily transport lithium ions and improve the ionic conductivity of the electrolytes. Furthermore, the organic building block of COFs facilitates the integration of functional groups, conferring various functional properties to electrolytes [28,34,35,36]. In summary, the potential applications of COFs in SSEs are highly promising, and they have spurred a significant increase in research interest as a new type of ionic conductor.

**Figure 1:**
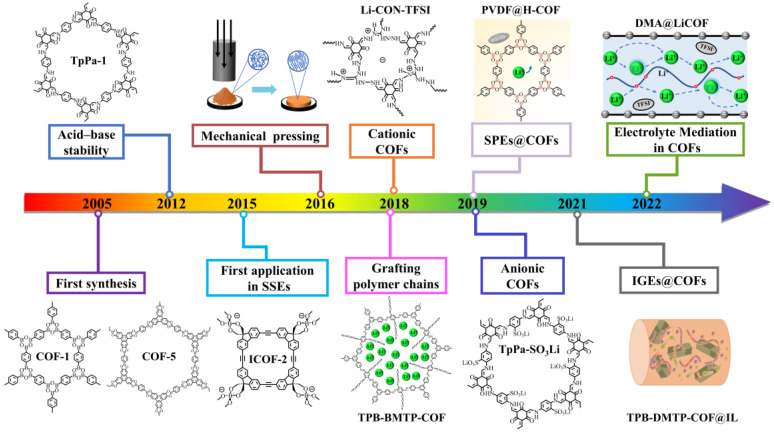
A brief chronology of the development of COFs in SSEs.

To concisely illustrate the development course of COFs in SSEs, some key events are summarized in Figure 1.

## 2. Design Principles and Structural Features of COFs

**Figure 2:**
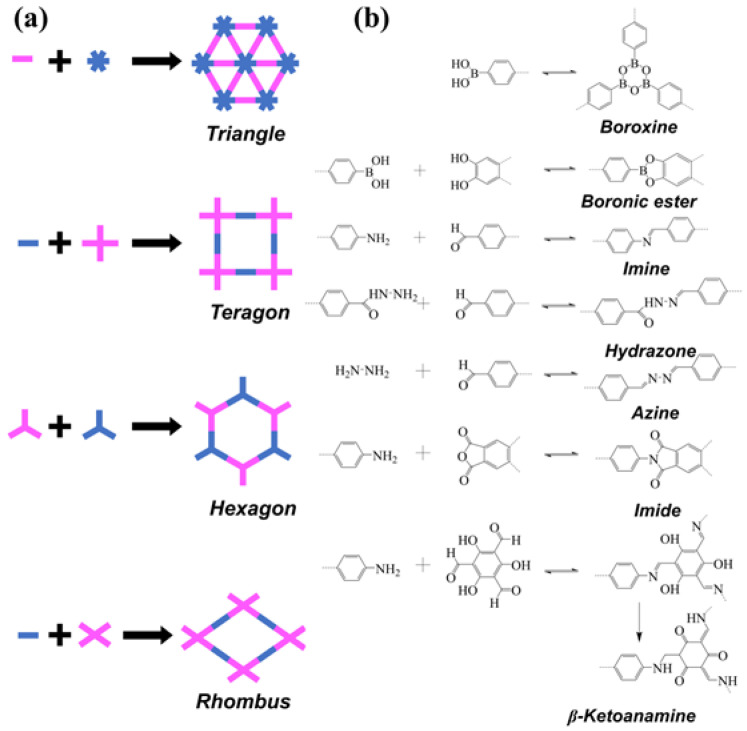
(**a**) The geometry of the building blocks of 2D COFs; (**b**) Different reactions for the formation of COFs.

To develop COF materials for high-performance electrolytes, it is crucial to have a thorough understanding of their design principles and structural features. All current COFs are constructed based on the concept of dynamic covalent chemistry (DCC), which is different from the kinetically controlled synthesis of conventional polymeric materials. This reversible formation of covalent bonds is thermodynamically controlled with “error checking” and “proof-reading” properties, resulting in the most thermodynamically stable structures [19]. The design principle of 2D COFs is based on the concept of reticulation chemistry. By using different building blocks, 2D COFs can produce shapes like triangles, teragons, hexagons, and rhombuses (Figure 2a), allowing for the regulation of pore shape, size, environment, and porosity through the use of symmetrical monomers. Additionally, COFs can be classified into different types, such as boroxines, boronic esters, imines, hydrazones, azines, and β-ketoenamines, based on the connection types of different covalent bonds [20] (Figure 2b). Although earlier COFs were not hydrostable [30,31], they have gradually become more stable in water and acid-base conditions with further development. For instance, TpPa-1 has become more chemically stable after undergoing irreversible enol-to-keto tautomerization [32]. The design principles of COFs allow the creation of materials with well-defined chemical structures in the backbone, adjustable pore size and geometry, and modified chemical and physical properties, making them promising candidates for SSEs.

COFs possess unique structural features that make them superior for solid-state electrolyte application, as outlined below.

**Figure 3:**
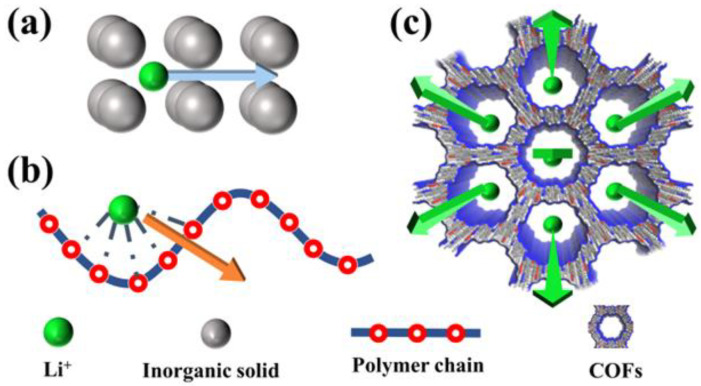
(**a**) Schematic diagram of the Li^+^ ion transport mechanism in (**a**) SIEs; (**b**) SPEs; (**c**) COFs.

(1) The ion conduction mechanism of COFs differs significantly from typical solid-state conductors [28]. In common SPEs, Li^+^ ion transport mainly relies on polymer-chain segmental motion, while in ISEs, it depends on ion hopping mechanism supported by defects in the lattice. However, the mechanism of Li^+^ ion transport in COFs is more complex [37]. COFs feature well-defined directional ion channels and consecutive ion-conducting sites. Within the nanopore channels, Li^+^ ions hop on the adjacent ion-conducting sites aligned in the axial pathway, achieving diffuse through ordered pores (Figure 3). This unique ion conduction mechanism of COFs effectively reduces the ion diffusion energy barrier and enhances Li^+^ transference number, resulting in excellent electrochemical performance.

(2) COFs have no glass transition temperature (Tg), avoiding the failure of most solid polymer electrolytes at low temperatures due to their high glass transition temperature [34]. This is because COFs contain a considerable number of covalent bonds in their molecular structure, which contributes to their highly ordered crystal structure, setting them apart from conventional polymers that generally exhibit amorphous structures. Additionally, COFs have exceptional thermal stability due to their strong covalent bonds. All of this makes them well-suited for application in batteries over a wide range of temperatures.

(3) The high cross-linking degree of COFs results in their insolubility in common organic solvents [38]. As solid-state electrolytes, COFs can substantially enhance the safety and stability of batteries, while also allowing for the incorporation of additional liquid electrolytes to further improve ionic conductivity.

(4) COFs are primarily composed of light elements. Their density is usually low. This presents a significant advantage in improving the gravimetric energy density of batteries, meeting the growing demand for higher energy densities.

(5) The porous structure of COFs enables them to accommodate small molecules and liquid electrolytes, while also exhibiting a unique nano-confinement effect that can lead to host-guest interactions [35].

(6) The high degree of crystallinity and long-range ordering exhibited by COFs significantly enhance their ability to facilitate ion transport. In comparison to conjugated microporous polymers (CMPs) [39], which belong to the same class of porous organic polymers [40], the reversible covalent bonds present in COFs contribute to their high crystallinity. Although CMPs also feature a highly cross-linked and porous structure, they are typically employed as electrode materials for lithium storage [41]. Due to the irreversible nature of CMPs constituent covalent bonds, resulting in an amorphous structure that is not conducive to ion transport. Conversely, the high level of crystallinity and ordered channels observed in COFs provide an effective pathway for the directional transport of lithium ions, rendering them more suitable as solid-state electrolyte materials.

(7) The organic nature of COFs enables the introduction of various functional groups into their skeletons, offering a wide range of functionalization possibilities, such as enhancing electrochemical stability, modifying ion conduction mechanisms, and regulating the solvation structure of lithium ions, among others.

The current applications of COFs in SSEs can generally be categorized into two aspects: COF-based electrolytes with COFs as matrix and composite electrolytes with COFs as fillers [28]. This paper will introduce the two major aspects and review the research progress of COF materials in solid SSEs of lithium metal batteries in recent years. Finally, the ion conduction strategies and design principles of COFs in SSEs are summarized and prospected.

## 3. COF-Based Electrolytes

### 3.1. Performance Optimizing Strategies for Electrolytes

When COFs are used as host materials of electrolytes, their pores can provide stable channels for lithium-ion transport. However, the secondary bonding, such as π-π stacking along the *z*-axis of COFs, leads to a larger interlayer spacing than that of inorganic crystals, so that the Li-ion binding sites are far apart. This is the disadvantage of rapid lithium-ion conduction [28]. In order to raise ionic conductivity to the level of commonly used non-aqueous electrolytes [42], the following strategies are usually proposed:

(1) Introducing solvent molecules and lithium salts into COFs. The unmodified, pure, electroneutral COFs do not inherently possess the ability to conduct lithium ions and, therefore, typically require the addition of lithium salts. The incorporation of a small number of organic solvents, such as propylene carbonate (PC), ethylene carbonate (EC), dimethyl carbonate (DMC), or diethyl carbonate (DEC), can significantly enhance both the ionic conductivity and the Li^+^ transference number. This is because solvents can act as plasticizers to promote ion migration. Moreover, polar solvents with high dielectric constants can promote the dissociation of weakly bound Li ions from the insoluble COF backbone, thus enabling fast ion conduction.

(2) Integrating flexible units or polymer chains onto the pore walls of COFs to provide hopping sites for Li^+^. COFs can be easily modified with various functional groups, allowing for the chemical anchoring of polymer chains to their pore walls. Furthermore, polymers and ionic liquids can be encapsulated into the pores of COFs by physical means due to their nano-confinement effect. This can greatly enhance the ion transport properties of the resulting materials.

(3) Inhibiting anion mobility or changing the coordination state of Li^+^ by constructing ionic backbones. Ionic covalent organic frameworks (iCOFs), which have permanently charged backbones, can be synthesized by assembling monomers with ionic groups or by post-functionalizing pre-assembled electroneutral COFs. Depending on the nature of their ionic groups, these materials can be classified as anionic or cationic COFs. Through functionalization, iCOFs can achieve synergy between different ionic conduction mechanisms. Anionic COFs, in particular, offer a distinct advantage in that they fundamentally avoid the need for lithium salts, making them a promising alternative for building solvent-free, all-solid COF-based electrolytes.

**Figure 4:**
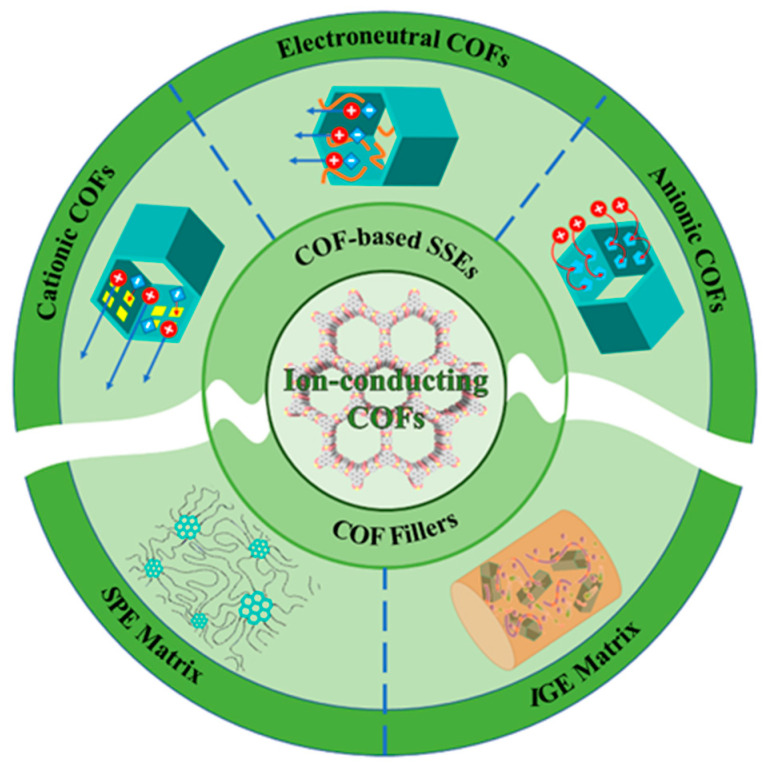
Schematics of applications for ion-conducting COFs in SSEs.

These strategies have derived three different charge types of COFs, including electroneutral, cationic, and anionic COFs (as shown in Figure 4) [35]. The research progress on each type of COFs in recent years will be introduced in the following sections.

### 3.2. Electroneutral COFs

For electroneutral COFs, as the host to accommodate guest molecules, the pore characteristics provide distinct nanoconfinement effects on COFs, which can affect the dynamic and thermal behavior of guest molecules to some extent. Therefore, when used to accommodate various lithium salts, organic solvents, polymer chains, and ionic liquids (ILs), COFs can overcome the shortcomings of traditional liquid electrolytes and solid polymer electrolytes.

**Figure 5:**
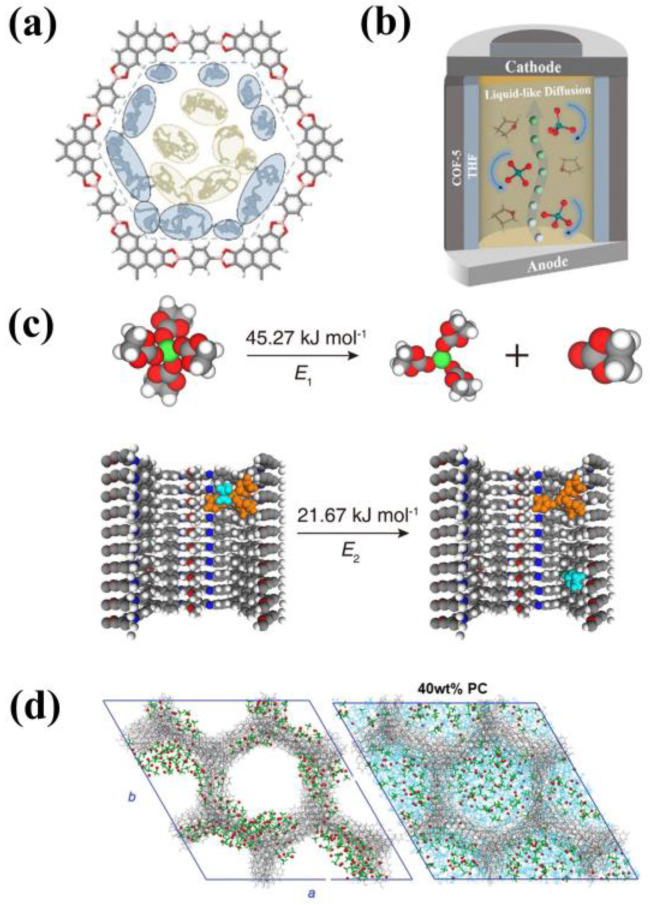
(**a**) Probability densities of the centers of THFs. Reproduced with permission from Ref. [44], Copyright (2019), Royal Society of Chemistry; (**b**) Schematic graphs of ion transport in COF−based SSEs; (**c**) DFT calculation model of Li(EC)4^+^ desolvation: in the pure liquid electrolyte and on the channel wall of TPB−DMTP−COF. Reproduced with permission from Ref. [45], Copyright (2020), Royal Society of Chemistry; (**d**) Snapshots at 200 ns for TAPB−PDA COF mixed with LiClO4 with 0 wt% and 40 wt% PC, Red spheres, green rods and cyan lines represent Li^+^, ClO4^−^ and PC molecules, respectively. Reproduced with permission from Ref. [46], Copyright (2022), Royal Society of Chemistry.

Generally, the 1D channels of COFs are randomly oriented, and their isotropy increases the tortuosity of ion transport paths, leading to lower ion mobility and diffusion flux [34]. In 2016, Uribe-Romo’s team prepared COFs with holistically crystallographic orientation and 1D ion transport channels through high-pressure mechanical pressing [43]. When COFs powder was impregnated in 1M LiClO4/THF, the ionic conductivity of COF-5 and TpPa-1 SSEs reached 2.6 × 10^−4^ S cm^−1^ and 1.5 × 10^−4^ S cm^−1^ at room temperature, respectively. Subsequently, in 2019, Pan’s team studied the LiClO4/THF-COF system with molecular dynamics simulation [44], and they found that THF molecules acted as lubricants and played a role in isolating Li^+^ and COFs (Figure 5a). Meanwhile, ClO4- and THF underwent free rotation and short-distance translation motion. As a result, Li^+^ diffusion performed a liquid-like behavior (Figure 5b). In 2020, He and Xu conducted experiments using TPB-DMTP-COF (TPB, triphenylbenzene; DMTP, dimethoxy terephthalaldehyde) as a separator, and LiPF6-EC as solvent [45]. It was found that COFs could adsorb Li^+^ solvates and anions, leading to a reduction in the desolvation energy of Li(EC)4^+^ (Figure 5c). In 2022, Li’s team utilized molecular dynamics simulations with electronic continuum correction to study the distribution and movement of Li^+^ in TAPB-PDA COF (TAPB: 1,3,5-tris(4-aminophenyl)benzene, PDA: terephthalaldehyde) [43]. Their research clearly demonstrated that LiClO4 tended to form clusters and attach to the pore walls of TAPB-PDA COF (Figure 5d). The addition of propylene carbonate (PC) faclitated the dispersion of Li^+^ in the pore of COFs, resulting in increased Li^+^ mobility. Based on these studies, it can be concluded that lithium salts and solvents do have effects on the Li^+^ mobility and diffusion flux in electroneutral COFs.

**Figure 6:**
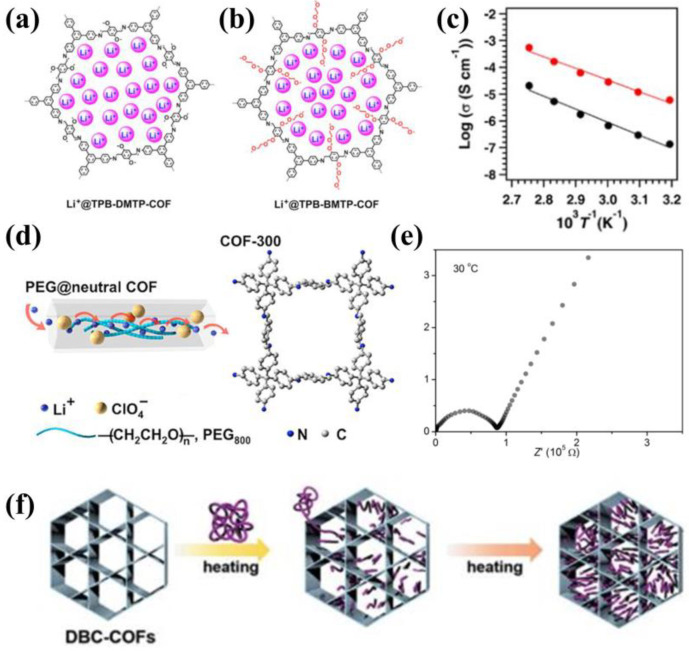
Electroneutral COFs host for Li−ion conduction (chemical anchoring): The structure of (**a**) Li^+^@TPB−DMTP−COF and (**b**) Li^+^@TPB−BMTP−COF; (**c**) Arrhenius plots of Li^+^@TPB−DMTP−COF (black) and Li^+^@TPB−BMTP−COF (red). Reproduced with permission from Ref. [47], Copyright (2018), American Chemical Society; (**d**) Schematic illustrations of Li^+^ transport in COF−300; (**e**) The EIS spectra of PEG−Li^+^@COF−300 at 30 °C. Reproduced with permission from Ref. [48], Copyright (2018), American Chemical Society; (**f**) Infiltration of PEG into nanochannels of DBC−2P. Reproduced with permission from Ref. [49], Copyright (2019), John Wiley and Sons.

With further advancements in COF-based electrolytes, recent research recognizes that grafting oligomer chains or introducing polymer chains onto COFs is a useful method to increase the ion mobility. In 2018, Jiang’s team reported the first example of grafting oligomer chains onto the pore walls of COFs [47]. They grafted methoxy and flexible oligo (ethylene oxide) chains onto the pore walls of COFs to synthesize TPB-DMTP-COF and TPB-BMTP-COF, respectively (Figure 6a,b). The oligo (ethylene oxide) chains formed a polyelectrolyte interface in the nanochannels, which facilitated the dissociation of lithium salts and provided a continuous migration channel for the transport of Li^+^. After impregnated with LiClO4, the ionic conductivity of the grafted COFs increased by three orders of magnitude more than bare channel COFs, reaching 6.04 × 10^−6^ S cm^−1^ at 40 °C (Figure 6c). In 2019, Wang and Feng introduced low-molecular-weight polyethylene glycol (PEG) into the channels of COFs (Figure 6d) [48]. Benefitting from the nanoconfinement effects of COFs, the room-temperature recrystallization of PEG was inhibited, and Li-ion transport was boosted efficiently. Accordingly, the ionic conductivity of PEG-Li^+^@COF-300 reached 1.40 × 10^−6^ S cm^−1^ at 30 °C (Figure 6e). In addition, the authors loaded PEG into cationic and anionic COFs to obtain higher ionic conductivity and Li^+^ transference number. It is demonstrated that non-electroneutral COFs might be more conducive to enhanced ionic conductivity, we will discuss this in more detail later. In the same year, Chen’s team synthesized the highly crystalline COFs (DBC-2P) and incorporated low-molecular-weight PEG and LiBF4 into the 1D nanochannel of DBC-2P (Figure 6f) [49]. The ionic conductivity of DBC-2P was as high as 2.31 × 10^−3^ S cm^−1^ at 70 °C.

**Figure 7:**
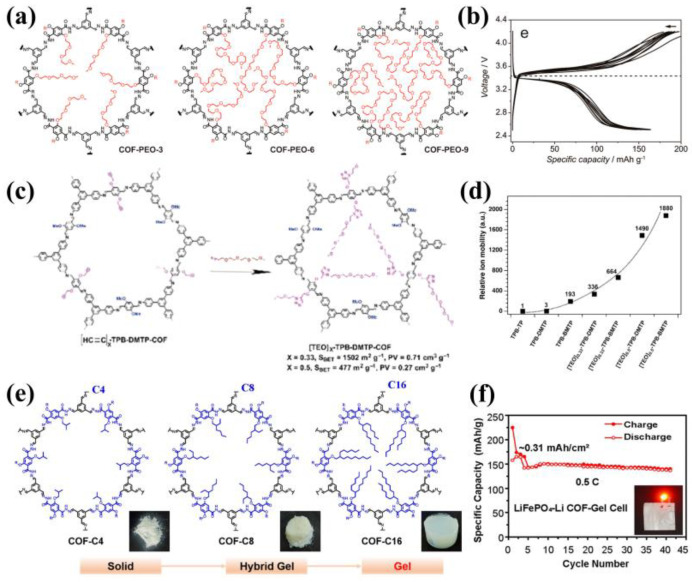
(**a**) Structure of COF−PEO−x (x = 3, 6, 9); (**b**) Charge-discharge curves of LiFePO_4_/COF-PEO−9−Li/Li cell at 100 °C. Reproduced with permission from Ref. [50], Copyright (2019), American Chemical Society; (**c**) Structure of [TEO]X−TPB−DMTP−COFs; (**d**) The relative ion mobility of Li^+^@TPB−TP−COF at 40 °C. Reproduced with permission from Ref. [51], Copyright (2020), John Wiley and Sons; (**e**) The structure of COF−Cx (x = 4, 8, 16); (**f**) Cycling performance of COF−gel−electrolyte in LiFePO4−Li full cells at 0.5 C. Reproduced with permission from Ref. [52], Copyright (2021), John Wiley and Sons.

The Introduction of polymer chains with different lengths may have different effects on ion conduction. In 2019, Horike and his colleagues studied the ionic conductivity of COFs modified by PEO chains with different lengths [50]. As shown in Figure 7a, COF-PEO-9-Li had the highest ionic conductivity, indicating that longer chain length had efficiently upgraded the ion conduction. However, a drawback of the LiFePO4/COF-PEO-9-Li/Li cell is its restricted operating temperature of 100 °C (Figure 7b). Jiang’s team integrated tetra (ethylene oxide) polyelectrolyte units (TEO; C_8_H_17_O_4_) into COFs pore walls in 2020 (Figure 7c) [51]. With the introduction of TEO chains, the relative ion mobility obtained a wide range of promotion (Figure 7d), which was consistent with Horike’s research [44]. COFs are mostly highly cross-linked crystalline powders, which are insoluble, infusible, and hard to process. In 2022, Zhang’s group prepared COFs gel electrolytes through introducing branched alkyl side chains with different lengths as internal plasticizers into COFs (Figure 7e), which greatly ameliorated the poor machinability of COFs. The assembled pouch battery can be stably cycled 41 times with a specific capacity of 136.5 mAh g^−1^, demonstrating the feasibility of COFs gel electrolyte (Figure 7f) [52]. It can be seen that the engineering of low molecular side chains or the introduction of polymer chains as mediation in COFs may effectively enhance the ionic conductivity of COFs and provide COFs good processability.

**Figure 8:**
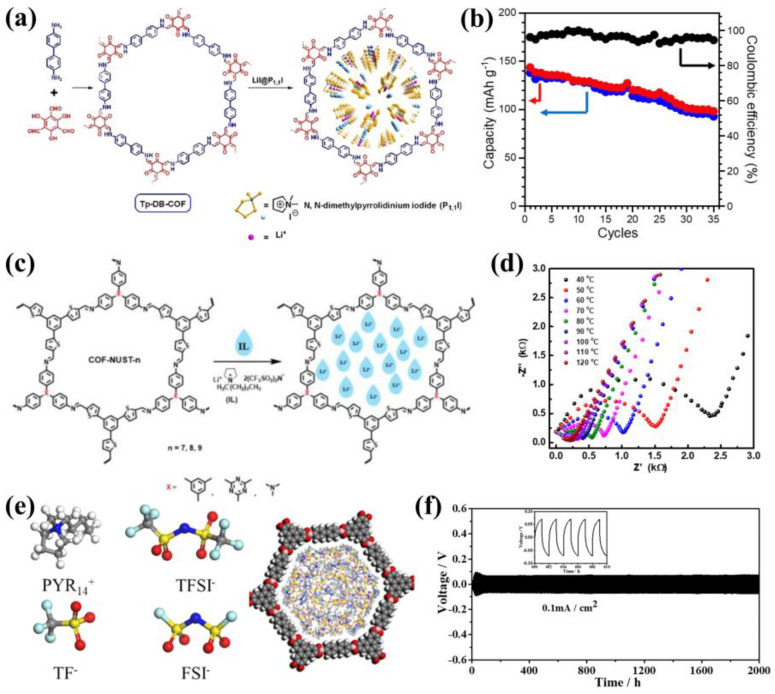
(**a**) Schematic view of LiI@P1,1I impregnated into COFs and chemical structures of P1,1I and Tp−DB−COF; (**b**) Cycling performances of LiFePO_4_|Li battery with LPC−2 electrolyte at 0.2 C. Reproduced with permission from Ref. [53], Copyright (2021), John Wiley and Sons; (**c**) The structure of COF−IL composite electrolytes; (d) Nyquist plots of IL−1.0@NUST−7 at different temperatures. Reproduced with permission from Ref. [54], Copyright (2021), American Chemical Society; (**e**) Structure models of COFs@ILs; (**f**) The voltage–time curves of the Li|COF@IL|Li cell at the current density of 0.1 mA cm^−2^. Reproduced with permission from Ref. [55], Copyright (2022), Elsevier.

In addition to combing with low molecular weight polymer chains, there are also other applications for electroneutral COFs. Wu et al. impregnated organic ionic plastic crystals (OIPCs) N,N-dimethylpyrrolidinium iodide (P1,1l) into the pores of COFs, and fabricated an innovative solid-state electrolyte (Figure 8a) [53]. The ionic conductivity of the composite electrolyte, Lil@P1,1l-2% COF (LPC-2), reached 4.36 × 10^−4^ S cm^−1^ at 60 °C, and the maximum specific capacity of LFP/Li full cell was 144.6 mAh g^−1^ at 0.2 C (Figure 8b). Furthermore, Zhang’s research group doped ionic liquid (IL) into 1D nanochannels of COFs in 2021, and then constructed three crystalline thiophene-based imine-linked COFs (COF-NUST-7/8/9) (Figure 8c) [54]. COF-NUST-7 had ionic conduction over a wide temperature range when IL/LiTFSI was 4:1 and (IL^+^LiTFSI)/COFs was 1:1 (Figure 8d). The LiFePO4-Li full cell also had a high initial discharge specific capacity of 140.8 mAh g^−1^ at 100 °C. In 2022, Sun’s team loaded ionic liquid and lithium salt into COFs channels to construct a quasi-solid electrolyte (Figure 8e) [55]. The study findings indicated that the risk of ionic liquid leakage is amplified in COFs with large pores, whereas COFs with smaller pore sizes tend to inhibit the diffusion of ionic liquid. The most optimal electrochemical performance was achieved with COFs that have medium-sized pores, which had the ionic conductivity of 8.7 × 10^−4^ S cm^−1^ at 30 °C and showed better lithium dendrite inhibition effect (Figure 8f).

To sum up, the current research on electroneutral COF-based solid electrolytes mainly focuses on the combination of COFs and polymers, including chemical anchoring and physical encapsulation. The rigid skeleton of COFs and its nanoconfinement effect ameliorate the deficiency of polymer ionic conductors, thus enhancing0 ion transport. However, the electroneutral COF-based electrolytes are dual-ion systems where both Li^+^ and its counter anions are mobile, leading toissues such as unfavorable Li^+^ transference number, concentration polarization and lithium dendrite [56]. To address these problems, it is essential to select a suitable polymer phase, ionic liquid, lithium salt, and COFs host to design a chemical environment that is more conducive to lithium single ion transport. Moreover, the design of non-electroneutral COF-based electrolytes has become an effective strategy to developing high-performance ionic conductors.

### 3.3. Cationic COFs

**Figure 9:**
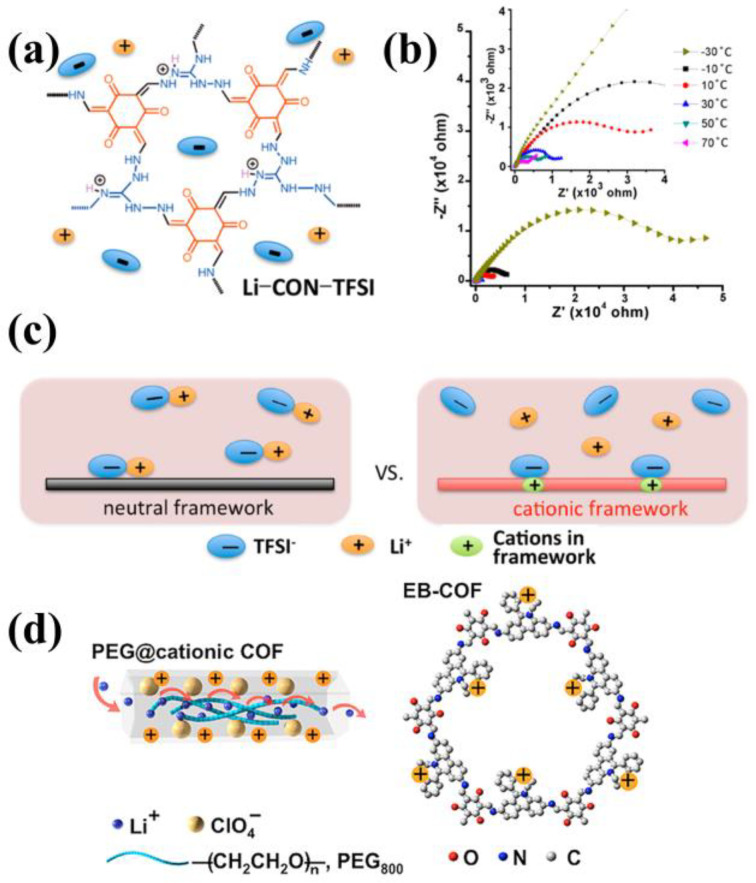
(**a**) Structure of the CONs with cationic framework for Li^+^ conduction; (**b**) EIS of Li−CON−TFSI at different temperatures; (**c**) Schematic illustrations of ion association in COFs with neutral and cationic frameworks, respectively. Reproduced with permission from Ref. [60], Copyright (2018), American Chemical Society; (**d**) Schematic illustrations of Li^+^ transport in EB−COF. Reproduced with permission from Ref. [48], Copyright (2018), American Chemical Society.

Ionic conductivity is the product of ion concentration and ion mobility [57]. Lithium salts in COFs exist as tightly associated ion pairs. Consequently, ion diffusion kinetics are sluggish and ionic conductivity is low [58]. Moreover, Li^+^ typically exhibits lower mobility than their counter anions, resulting in Li^+^ transference number typically lower than 0.5. This scenario is exacerbated by anion accumulation on the surface of lithium metal electrodes, which promotes detrimental interface side reactions, and concentration polarization during battery discharge cycles, and then resulting in voltage losses, higher internal impedance, and ultimately, cell failure [59]. To address this issue, cationic COFs can be developed to immobilize lithium salt anions onto the COF framework, facilitating single Li^+^ conduction in solid electrolytes. In 2018, Chen et al. introduced a cationic group into the COFs skeleton, and separated Li-ion pairs by stronger dielectric screening, finally preparing solvent-free high ionic conduction materials [60]. They synthesized cationic COFs nanosheets using Triaminoguanidinium chloride (TGCl) and 1,3,5-Triformylphloroglucinol (Tp), then mixed with LiTFSI after anion exchange to obtain Li-CON-TFSI (Figure 9a). The Li^+^ transference number of Li-CON-TFSI was 0.61, and the ionic conductivity reached 5.74 × 10^−5^ S cm^−1^ at 30 °C and 2.09 × 10^−4^ S cm^−1^ at 70 °C (Figure 9b). Compared with the electroneutral COFs, the cationic skeleton can more interact with TFSI- through electrostatic force, thus releasing Li^+^ (Figure 9c), and obtaining excellent ionic conduction. In addition, low-molecular-weight polymer was also introduced into cationic COFs to promote ion conduction. In 2019, Wang and Feng’s group introduced low-molecular-weight PEG into cationic COFs [48], and infiltrated lithium salt to construct PEG-Li^+^@EB-COF-ClO4 (Figure 9d). The ionic conductivity of PEG-Li^+^@EB-COF-ClO4 reached 1.93 × 10^−5^ S cm^−1^ at 30 °C, which was higher than that of electroneutral COFs and was the highest value among solid-state conductors of known solvent-free polymer crystalline porous materials at that time. The Li^+^ transference number of PEG-Li^+^@EB-COF-ClO4 is also as high as 0.60 due to its cationic skeleton.

**Figure 10:**
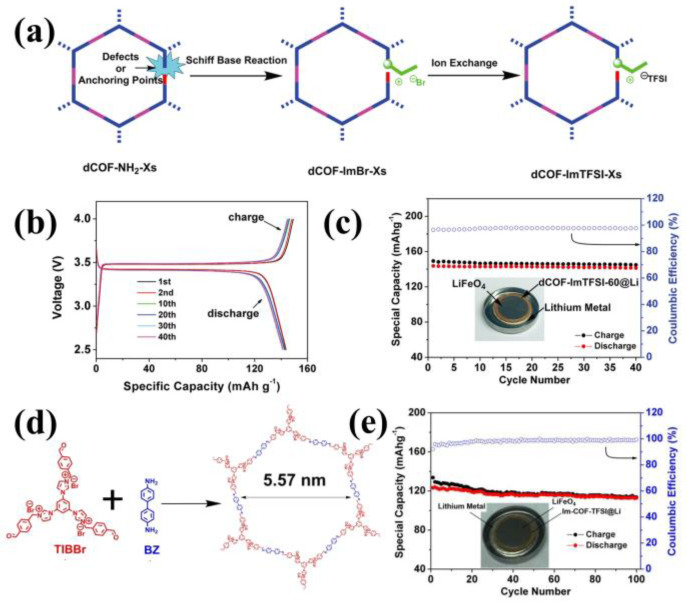
(**a**) Synthesis of dCOF−ImTFSI via ion exchange method; Charge−discharge curves (**b**) and cycling performance (**c**) of Li/dCOF−ImTFSI@Li/LiFePO_4_ at 0.1 C under 80 °C. Reproduced with permission Ref. [63], Copyright (2020), John Wiley and Sons; (**d**) Synthesis of Im−COF; (**e**) cycling performance of the Li/Im−COF−TFSI@Li/LiFePO_4_ at 0.1 C at 80 °C. Reproduced with permission from Ref. [64], Copyright (2020), Royal Society of Chemistry.

Ionic liquids with imidazolium cations tended to show higher ionic conductivity [61,62]. As motivation, Han et al. introduced imidazolium into defective COFs by post-functionalization in 2020 [63]. They manufactured 2D-COFs (dCOFs) by introducing defects, then substituted the imidazolium functional group for the active amine through post-functionalization, and then exchanged the Br- in imidazolium with TFSI (Figure 10a) to obtain dCOF-ImTFSI-Xs (X = 20, 40 and 60). With the maintained porous structure and crystallinity after post functionalization, the as-prepared dCOF-ImTFSI-60@Li showed the ionic conductivity of 9.74 × 10^−5^ S cm^−1^ and the Li^+^ transference number of 0.72 at 30 °C. These results indicated that the interaction between charged imidazolium functional groups and lithium salts could facilitate ion pair dissociation, and the highly delocalized TFSI- was conducive to higher ionic conductivity, resulting in good battery performance (Figure 10b,c). In the same year, Han et al. also integrated imidazolium directly into COFs. They synthesized cationic imidazolium-based COFs (Im-COF) through the condensation reaction by using 1,3,5-tris [3-(4-formylbenzyl)-1H-imidazol-1-yl]benzene bromide (TIBBr) as cationic building block and benzidine (BZ) as neutral unit [64] (Figure 10d). Finally, by exploiting the ion-exchange strategy, solid state Li^+^ conductors Im-COF-TFSI@Li was prepared. Its ionic conductivity and Li^+^ transference number were 2.924 × 10^−5^ S cm^−1^ and 0.62 at 30 °C, respectively. The assembled LiFePO4|Li full battery delivered an initial specific capacity of 123.3 mAh g^−1^ at 80 °C and 0.1 C, and the capacity retention reached 91.6% after 100 cycles (Figure 10e).

However, the cationic COFs-based electrolytes also have obvious deficiency. The synthesis process requires the addition of a large amount of extra lithium salts. This is necessary not only to introduce lithium salt anions in advance to balance the charge of the cationic skeleton but also to provide sufficient Li^+^ for ion transport. As a result, some lithium salt anions exist as counter ions of the cationic skeleton, while others remain as ion pairs, consequently, excessive lithium salt anions are inevitable.

### 3.4. Anionic COFs

In order to eliminate the mobility of Li salt anions and develop a single Li-ion conducting solid electrolyte, researchers have attempted to graft weakly coordinated organic anion groups, such as borate, imidazolate, carboxylate and sulfonate anions, directly onto the COFs skeleton to construct anionic COFs. The resulting negatively charged frameworks provide hopping sites for the migration of Li^+^ and have shown great application potential.

**Figure 11:**
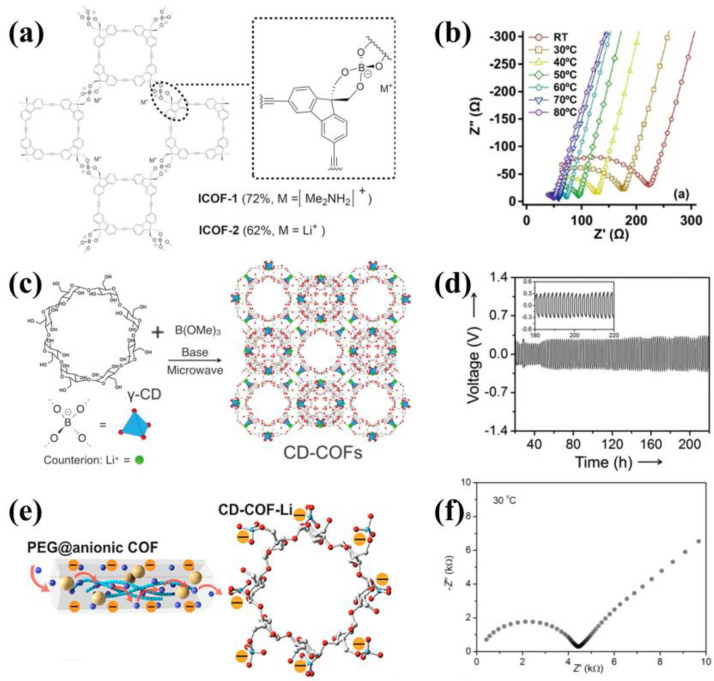
(**a**) Structure of ICOF−1 and ICOF−2; (**b**) Nyquist plot of ICOF−2. Reproduced with permission from Ref. [65], Copyright (2015), John Wiley and Sons; (**c**) Synthesis of CD−COFs; (**d**) Stripping/plating test of Li/CD−COF−Li@LiPF_6_−EC−DMC/Li at a current density of 0.085 mA cm^−2^.Reproduced with permission from Ref. [66], Copyright (2017), John Wiley and Sons; (**e**) Schematic illustrations of Li^+^ transport in CD−COF−Li; (**f**) The EIS spectra of PEG−Li^+^@CD−COF−Li at 30 °C. Reproduced with permission from Ref. [48], Copyright (2018), American Chemical Society.

In 2016, spiroborate-linked anionic COFs were reported by Zhang and his colleagues, which is the first example of COF-based SSEs [65]. They constructed novel ionic COFs with sp3 hybridized boron anionic centers and tunable counter-cations (ICOF-1 and ICOF-2, Figure 11a). Unlike the hydrolysis-prone boronate ester linkages, the ICOFs showed favorable stability in water due to the additional coordination effect of Lewis base with boron. ICOF-2 with 55% propylene carbonate obtained an ionic conductivity of 3.05 × 10^−5^ S cm^−1^ (Figure 11b) and a high Li^+^ transference number value of 0.80 at room temperature. In 2017, Wang et al. synthesized a 3D anionic COF based on γ-cyclodextrin (γ-CD) molecules via microwave-assisted solvothermal methods [66] (Figure 11c). The CD-COFs film in 1M LiPF6-EC-DMC revealed an ionic conductivity of 2.7 × 10^−3^ S cm^−1^ at 30 °C and an activation energy of 0.26 eV. The Li symmetrical cell was cycled over 220 h at a current density of 0.085 mAcm^−2^ (Figure 11d), showing a promising Li^+^ stripping/plating stability. In 2019, they also proposed a strategy of incorporating PEG into CD-COF-Li to accelerate Li^+^ conduction [48] (Figure 11e). The CD-COF-Li loaded LiClO4 and PEG without any solvents obtained an ionic conductivity of 2.6 × 10^−5^ S cm^−1^ at 30 °C (Figure 11f).

**Figure 12:**
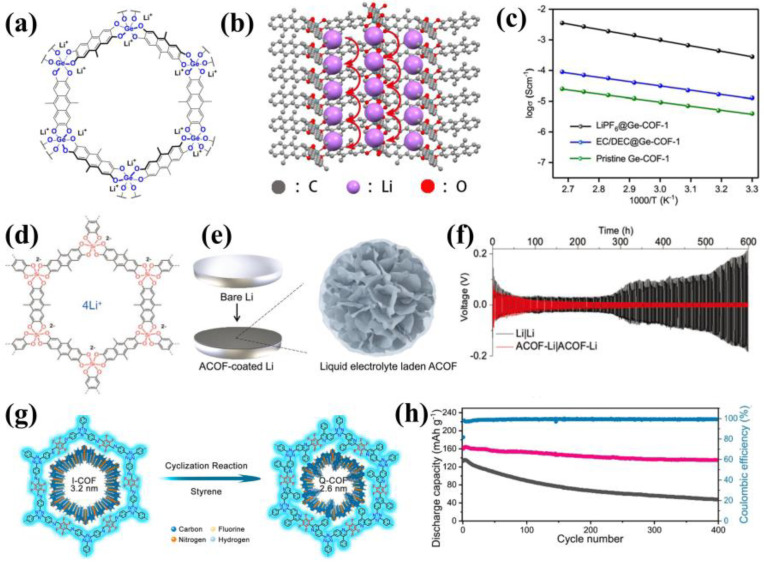
(**a**) Structure of germanate covalent organic framework Ge−COF−1; (**b**) Side view of the channel and potential Li−ion hopping pathway along the channel; (**c**) Arrhenius plots of different Ge−COF. Reproduced with permission from Ref. [68], Copyright (2019), John Wiley and Sons; (**d**) Schematic illustration shows ACOF−coated Li, where the ACOF is composed by particles of 2D nanosheets that are laden with liquid electrolyte; (**e**) Structural representation of ACOF; (**f**) Galvanostatic cycling of Li symmetric cells at 1 mAh cm^−2^ and 1 mA cm^−2^. Reproduced with permission from Ref. [69], Copyright (2021), John Wiley and Sons; (**g**) Synthesis of Q−COF by the cyclization reaction of I−COF; (**h**) Cycling performance of Li|Q−COF|NMC811 cells with Q−COF prepared at high temperature (pink) and RT (black). Reproduced with permission from Ref. [66], Copyright (2021), John Wiley and Sons.

Motivated by the intuition that heavier elements could improve ionic conductivity [67], Wang’s team introduced heavier elements into COFs [68]. They synthesized germanate anionic COFs (Ge-COF-1) by the combination of a hex-coordinated germanate center with a tetrahydroxylated anthracene via microwave heating (Figure 12a). In Ge-COF-1, free Li^+^ could hop along the channel in the skeleton (Figure 12b). Introducing heavier elements into COFs not only increased the pore width but also reduced the affinity for ions, which provided more freedom for Li^+^ to flow in the channels. Consequently, LiPF6@Ge-COF-1 showed a significantly improved ionic conductivity of 2.5 × 10^−4^ S cm^−1^ and a Li^+^ transference number value of 0.67 at 30 °C (Figure 12c). In 2021, Lu et al. constructed hexacoordinated silicate anionic COFs (ACOF) [69] with a chemical structure similar to Ge-COF-1 (Figure 12e) and used them as electrolyte interphase to protect Li metal anode. The Li^+^-affinity and layered structure of ACOF facilitated rapid Li^+^ conduction (Figure 12e). And the high Li^+^ transference number (0.82) and ionic conductivity beyond 3.7 mS cm^−1^ could be achieved when soaked in 1M LiPF_6_ in EC/DEC. The adsorption of Li^+^ by ACOF led to the localization and decomposition of associated anions from the electrolyte, forming stable SEI and dendrite-free morphology. And pairing the ACOF-coated Li against a high-voltage LiCoO_2_ cathode (4.5 V) achieved exceptional cycling stability (Figure 12f). In 2021, Xu’s team designed an original type of quinolyl-linked COFs (Q-COFs), they successfully constructed high-voltage-tolerant SSEs by introducing lithiophilic groups and electrochemically stable quinolyl aromatic ring linkages [70]. They first synthesized highly crystalline imine-linked COFs (I-COFs) by solvothermal method, and further obtained quinolyl-linked COFs (Q-COFs) through the Povarov reaction (Figure 12g). After soaking degassed Q-COFs into 1 M LiTFSI/THF and mechanical pressing at high temperatures, they obtained flexible SSE films with holistically crystallographic orientation and a large Young’s modulus of 10.5 GPa. Besides, the films showed favorable ionic conductivity (7.5 × 10^−5^ S cm^−1^) and a large Li^+^ transference number (0.72) at room temperature. Q-COF exhibited a large band gap with an ultralow HOMO value (−6.2 eV under vacuum) and oxidative stability up to 5.6 V, which manifested good potential in high-voltage batteries. And the NCM811||Q-COF||Li batteries showed a stable cycling performance with coulombic efficiency of 99% and capacity retention of 82% after 400 cycles (Figure 12h).

**Figure 13:**
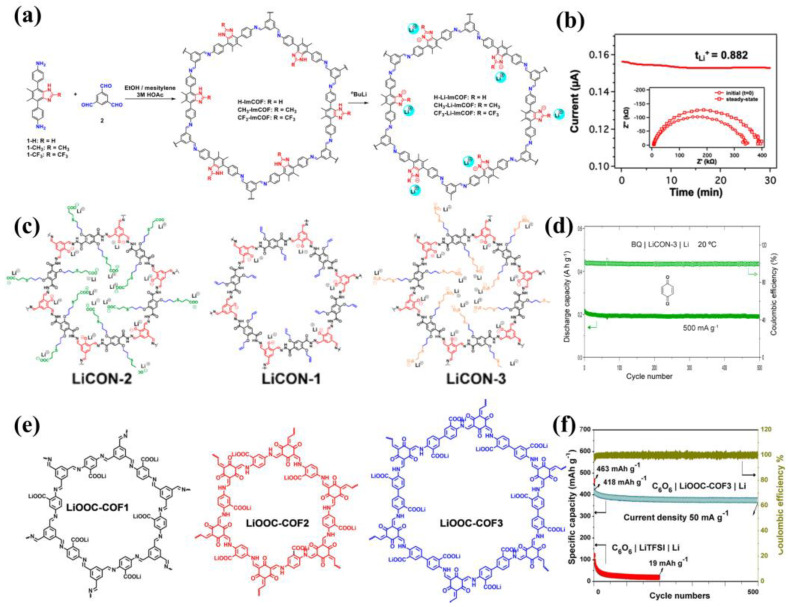
(**a**) Synthesis and structure of Li−ImCOFs; (**b**) lithium transference number calculated using the Bruce−Vincent−Evans technique. Reproduced with permission from Ref. [71], Copyright (2019), American Chemical Society; (**c**) Structure of LiCON; (**d**) Cycling performance of BQ|LiCON−3|Li cells. Reproduced with permission from Ref. [72], Copyright (2020), American Chemical Society; (**e**) Structure of LiOOC−COF; (**f**) cycling performance of the C_6_O_6_|LiOOC−COF_3_|Li battery at a current density of 50 mA g^−1^ [73]. Ref. [73] is an open-access article distributed under the terms of the Creative Commons CC BY license, which permits unrestricted use, distribution, and reproduction in any medium provided the original work is properly cited.

The different substituents in anionic COFs have different effects on ion transport. In 2019, the first example of crystalline imidazolate-containing COFs was reported by Zhang’s team [71]. They synthesized a series of single-ion conducting COFs by varying the substituents (H, CH3, CF3) on imidazolate backbones and a simple lithiation with n-BuLi (Figure 13a). Upon solvation with ∼20 wt% PC, CF3-Li-ImCOF displayed a high Li^+^ transference number (0.81) (Figure 13b) and remarkable ionic conductivity (7.2 × 10^−3^ S cm^−1^) at room temperature with an activation energy of 0.10 eV. Thanks to the strong electron-withdrawing effect of CF3 substituent, the negative charge of imidazolate anions was delocalized and ion pairing was weakened, leading to high ion conductivity. In 2020, Loh and his colleagues reported single-ion conducting COFs (LiCON-x) with wide-temperature applicability [72]. They introduced anion groups into COFs as the end groups of flexible long chains, which was synthesized by one-pot solution and treated with aqueous lithium carbonate to get lithiated covalent organic nanosheets (LiCONs) after washing and drying (Figure 13c). The COFs with more acidity of anions showed higher ionic conductivity. Therefore, LiCON-3 with sulfonate groups exhibited the best electrochemical properties. The solvent-free LiCON-3 displayed a large Li^+^ transference number (0.92) and superior ionic conductivity (3.21 × 10^−5^ S cm^−1^) at 20 °C with an activation energy of 0.13 eV and good ionic conductivity (0.9 × 10^−5^ S cm^−1^) even at −40 °C. The Li|LiCON-3|Li symmetric cells could stably cycle for 500 h at relatively high current densities of 0.05 and 0.1 mA cm^−2^. Using 1,4-Benzoquinone (BQ) as cathode, the assembled BQ|LiCON-3|Li organic batteries could run for 500 cycles at a current density of 500 mA g^−1^ with 87.7% retention of initial capacity (Figure 13d). In 2022, Guo’s team designed and prepared a series of single-ion conducting COFs regulated by Li-carboxylate [73]. Three Li-carboxylate COFs (denoted LiOOC-COFn, *n* = 1, 2, and 3) were obtained by soaking the different carboxylic acid functionalized COFs condensed by different ligands in Li_2_CO_3_ (Figure 13e). Among them, LiOOC-COF3 with ketone-form structure, rich Li-ion content, and larger pore size (1.9 nm) exhibited the best electrochemical performance, an exceptional ion conductivity of 1.36 × 10^−5^ S cm^−1^ at room temperature and a high transference number of 0.91. In addition, they used the organic carbonyl molecule cyclohexanehexone (C_6_O_6_) as a cathode and Li metal as an anode to assemble the C_6_O_6_|LiOOC-COF_3_|Li solid cells, which showed better cycling and rate performance than liquid electrolyte (Figure 13f).

**Figure 14:**
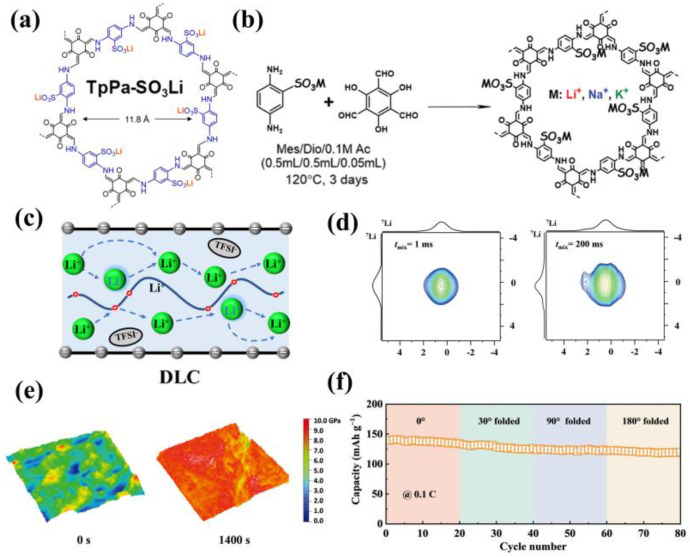
(**a**) Chemical structure of lithium sulfonated COF (TpPa−SO_3_Li). Reproduced with permission from Ref. [74], Copyright (2019), American Chemical Society; (**b**) Illustration of the pre−synthetic strategy for single−ion (Li^+^, Na^+^, K^+^) conductive COFs. Reproduced with permission from Ref. [75], Copyright (2020), Royal Society of Chemistry; (**c**) Illustration of the Li^+^ transport mode in DLC; (**d**) 2D 7Li–7Li exchange spectra measured at 298 K for DLC at t_mix_ = 1 ms and t_mix_ = 200 ms; (**e**) Quantitative nanomechanical mappings for lithium metal at sputtering times of 0 s and 1400 s; (**f**) Cycling performance of a Li|DLC|LFP pouch cell at various degrees of bending deformation at 45 °C. Reproduced with permission from Ref. [76], Copyright (2022), John Wiley and Sons.

A novel solvent-free lithium sulfonated COF (TpPa-SO_3_Li) was reported by Lee’s team in 2019 [74]. The hexagonal pore arrays with keto-enamine linkages were vertically stacked, sulfonates were tethered to the skeleton for single Li-ion conduction, and monoaromatic building blocks formed small pores to afford a high number density of Li-ions (Figure 14a). After cold pressing, TpPa-SO_3_Li exhibited a solvent-free ionic conductivity of 2.7 × 10^−5^ S cm^−1^ with a high transference number of 0.90 at room temperature. In 2020, Li et al. proposed a pre-synthetic strategy for the construction of lithium sulfonate COFs (Tp-PaSO_3_Li-COFs) and obtained single-ion conductors of different alkali metals (Li^+^, Na^+,^ and K^+^) [75]. Alkali metal ions were integrated to 2,5-diaminobenzenesulfonic acid by the acid-base reaction, and then condensed with aldehyde in a solvothermal environment to synthesize single-ion conducting TpPa-SO_3_Li-COFs (Figure 14b). When 10 mL EC/DMC (*v*/*v* = 1:1) was added to TpPa-SO_3_Li-COF as a plasticizer, it achieved outstanding ionic conductivity of 2.7 × 10^−5^ S cm^−1^, a high transference number of 0.94 and an activation energy of 0.13 eV at 20 °C. Furthermore, authors investigated the performance of LiFePO_4_||Tp-PaSO_3_Li-COF||Li cells, which presented the first cycle capacity up to 152 mAh g^−1^, but the battery capacity decayed rapidly. In 2022, a strategy of electrolyte mediation was reported by Manthiram’s team (Figure 14c) [76]. They entrapped the N,N-Dimethylacrylamide (DMA) and lithium salt (LiTFSI) into the pores of TpPa-SO_3_Li-COFS by a low-pressure-driven method, followed by in situ polymerization, and successfully prepared a flexible DMA@LiTFSI-mediated COF (DLC) electrolyte with high performance. LiTFSI could trigger the copolymerization of DMA solvent and provide additional Li^+^. The flexible DMA chains could not only release Li^+^ from the rigid COF backbone and simultaneously decouple the lithium salt, but also serve as a bridge for the hopping of Li^+^ (Figure 14d). The DLC electrolyte achieved outstanding electrochemical performance due to the novel way of Li^+^ transport. It showed ionic conductivity of 1.65 × 10^−4^ S cm^−1^, electrochemical window of 4.5 V, and Li^+^ transference number of 0.85 at 23 °C. According to the peak force quantitative nanomechanical mappings (PFQNM), a dual solid electrolyte interface (SEI) was formed on the surface of lithium metal anode, which included an inorganic inner layer that could efficiently suppress dendrite growth and an organic outer layer that could hold the electrode/electrolyte contact (Figure 14e). The LiFePO_4_||DLC||Li cells showed a high capacity of 145 mAh g^−1^ at 0.1 C, and a capacity of 120 mAh g^−1^ at room temperature during rigorous folding tests (Figure 14f). This state-of-the-art electrolyte mediation strategy paved the way toward more efficient single-ion conductors.

A great number of studies have demonstrated that the construction of anionic COFs is an effective strategy for the preparation of high-performance ionic conductors. However, the effects of the type, density and spatial arrangement of anionic groups on ion conduction need to be further discussed, and more attention should be paid to molecular computational simulation and ion transport mechanism to gain a more comprehensive understanding in the future studies.

The properties of various COF-based SSEs are summarized in Table 1.

It must not be overlooked that the use of COFs as electrolyte matrices presents several challenges. One of the main issues is the laborious preparation process. The synthesis based on dynamic covalent chemicals is heavily reliant on specific reaction conditions, such as temperature, time, and catalysts, and even slight variations can have a significant impact on the synthesis results. Additionally, the growth and crystallization of COFs occur simultaneously during the synthesis process, making it difficult to produce large quantities of highly crystalline and structurally stable COFs. Typically, the synthesis of COFs requires a minimum of 72 h at a temperature of 120 °C and the raw materials are also costly, resulting in high manufacturing costs. Another obstacle is the insolubility of COFs, which complicates their processing. Currently, COF-based electrolyte films are mostly constructed by powder pressing, which requires high pressure and sometimes heat treatment, further impeding the scale production of COF-based electrolytes. Furthermore, as shown in Table 1, there are few examples of COF-based electrolytes that have been successfully applied to full batteries in the early stages of development. Despite various strategies having been derived in later stages, significant breakthrough in battery performance remains elusive.

In summary, the current research on COF-based SSEs is continually advancing, with particularly focus on developing solvent-free solid electrolytes. The distinctive features of COFs can be utilized as a filler in the substrate to overcome some of the inherent limitations. The quantity of filler can be regulated, and even a small amount can yield satisfactory outcomes. Thus, research on utilizing COFs as a filler has become a promising avenue to explore.

## 4. Composite Electrolytes with COFs as Fillers

The poor processing performance of COFs hinders their further development, incorporating COFs into polymer matrices or ionic liquids to create composite electrolytes by casting and tape-casting offer a potential solution. Moreover, the nanopores and ordered channels of COFs can effectively enhance the efficiency of ion transport. and the rigid skeleton also provides certain mechanical properties. Therefore, COFs also demonstrate great potential as fillers.

**Figure 15:**
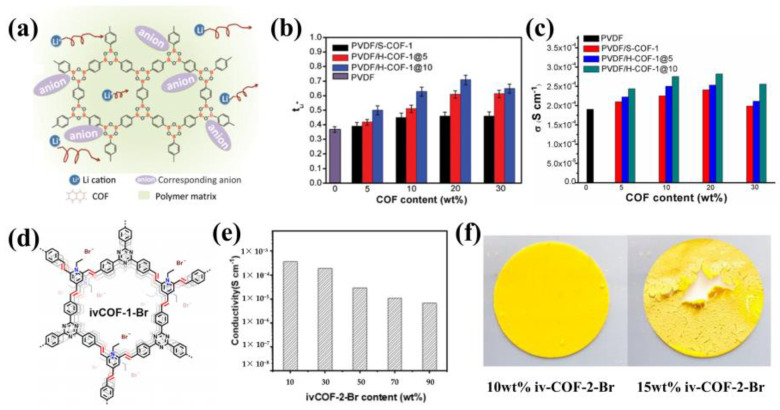
(**a**) Schematic diagram representing the anion adsorption capacity of boron−containing COFs; (**b**) Ionic conductivity of the electrolytes with different COF contents; (**c**) tLi^+^ of the electrolytes with different COF contents. Reproduced with permission from Ref. [77], Copyright (2019), Royal Society of Chemistry; (**d**) Structure of ivCOF−1−Br; (**e**) Conductivity of the composite pellets of Li/PEO@ivCOF−2−Br with COF contents from 10 wt% to 90 wt%; (**f**) Digital photographs of the composite films Li/PEO@ivCOF−2−Br with 10 wt% and 15 wt% ivCOF−2−Br contents, respectively. Reproduced with permission from Ref. [79], Copyright (2021), John Wiley and Sons.

The addition of COFs as fillers is highly effective in increasing the Li^+^ transference number of electrolytes due to their unique structure and functional groups. In 2019, Chen’s team reported the successful addition of boron-containing COFs (H-COF-1) into PVDF-based electrolyte to achieve significantly enhanced Li^+^ transference number [77]. They synthesized H-COF via a simple, template-free approach, and prepared PVDF gel composite electrolyte containing LiClO4 and COF additives by a solution casting technique (Figure 15a). Due to the strong anion adsorption capacity of boron-containing skeleton, freer Li^+^ are generated, and the high porosity and surface area of COFs provide abundant anion adsorption sites. As a consequence, the addition of COFs resulted in a significant increase in Li^+^ transference number compared to that of pure PVDF electrolyte (0.36). This increase was related to the specific surface area of COFs, and H-COF-1@10 with the largest specific surface area made the Li^+^ transference number ascend to 0.71 (Figure 15b). In addition, with 20 wt% of H-COF-1@10, the composite electrolyte exhibited an enhanced room-temperature ionic conductivity of 2.8 × 10^−4^ S cm^−1^ (Figure 15c). And the further increasing number of COFs would lead to a decrease in conductivity due to poor dispersion and resultant interference with the ion transport path. LiFePO_4_||PVDF/H-COF-1@10||Li full-cell batteries were assembled and cycled for 80 times at room temperature, with an initial capacity of 128 mAh g^−1^ and a capacity retention rate of 94%. However, it is worth noting that the water instability of boron-based COFs must be considered for practical applications [78].

The presence of nanochannels in COFs is a significant advantage in their use as electrolyte fillers. The ordered nanochannels provide improved ionic conductivity and unique nanoconfinement effects. Zhang and co-workers synthesized multiple vinylene-linked ivCOF-Br in 2021 (Figure 15d) and introduced COFs and LiTFSI into PEO by solution casting to fabricate flexible composite films [79]. The introduction of multiple ivCOF-Br synthesized by different ligands could increase the ionic conductivity of PEO matrix, as the crystallinity of PEO within the pores was reduced by the nanoconfinement effects of COFs. Particularly, when adding 10 wt% of ivCOF-2-Br, the ionic conductivity of the composite electrolyte climbed to 3.84 × 10^−4^ S cm^−1^ at 20 °C. While as the content of COFs continued to increase, the ionic conductivity tended to decrease (Figure 15e) due to the inconsecutive and fragile texture (Figure 15f), which is consistent with the previous findings.

**Figure 16:**
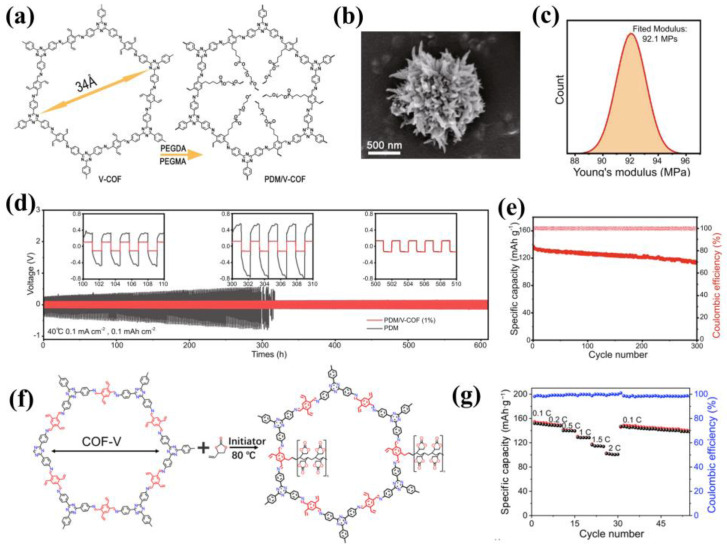
(**a**) Structure of V−COF and PDM/V−COF; (**b**) SEM image of V−COF; (**c**) Young’s modulus of PDM/V−COF (1%) after Gaussian fitting; (**d**) Long−term cycling of symmetrical lithium metal cells using PDM/V−COF (1%) (red) and PDM electrolyte (black) at 40 °C; (**e**) The long−time cycling performance of Li|PDM/V−COF (1%)|LiFePO_4_ cells at 1 C [80]. Ref. [80] is an open−access article distributed under the terms of the Creative Commons CC BY li−cense, which permits unrestricted use, distribution, and reproduction in any medium provided the original work is properly cited; (**f**) Structure of COF−PVEC composite electrolyte; (**g**) Rate performance of LFP/COF−PVEC/Li cells at varied current densities. Reproduced with permission from Ref. [81], Copyright (2022), Springer Nature.

The organic properties of COFs can be fully utilized as fillers in electrolytes. For instance, polymer chain segments can be grafted onto COFs to achieve better compounding of COFs and polymer phases. In 2022, Xu et al. prepared vinyl-functionalized COFs (V-COF) by room temperature synthesis, and grafted ether-based segments (PEGDA and PEGMA) onto COFs via in situ polymerization (Figure 16a) for the preparation of high-performance composite electrolytes (PDM/V-COF) [80]. The ether-based segments were successfully incorporated into the channels of the urchin-like V-COF (Figure 16b), which effectively decreased the crystallinity and the glass transition temperature of the polymer and provided an ordered channel for ion transport. When 1 wt% V-COF was uniformly dispersed in PDM, the PDM/V-COF composite electrolyte showed the best electrochemical performance, with the ion conductivity of 1.1 × 10^−4^ S cm^−1^ at 40 °C, the Li^+^ transference number of 0.45, and the electrochemical window as high as 5.0 V. Compounding PDM with COFs by chemical bonds could enhance the mechanical properties of the composite electrolytes, and its Young’s modulus reached 92 MPa, which is twice that of pure PDM (Figure 16c). Moreover, the triazine rings of V-COF facilitated the uniform lithium deposition, and Li symmetrical cells achieved stable cycles for more than 600 h at 0.1 mA cm^−2^ with a Li plating/stripping capacity of 0.1 mAh cm^−2^ (Figure 16d). The LiFePO4|PDM/V-COF (1%)|Li cells showed the initial capacity of 136 mAh g^−1^ at 1C with a high-capacity retention of 83.8% over 300 cycles (Figure 16e). In addition, Xu et al. also introduced the V-COF filler into the polyethylene carbonate (PVEC) electrolyte [81] (Figure 16f). When 2 wt% V-COF was added, the ionic conductivity of the composite electrolytes ascended to the highest (1.11 × 10^−4^ S cm^−1^ at 40 °C), which is two orders of magnitude higher than that of pure PVEC, the ion transference number reached 0.6, and the electrochemical window was up to 4.6 V. In terms of mechanical properties, Young’s modulus of the composite electrolyte was as high as 150 MPa, much higher than the 27 MPa of pure PVEC, which could effectively inhibit the lithium dendrites, resulting in the plating/stripping stability of Li symmetrical cell for 600 h at 0.1 mA cm^−2^. The LiFePO4|COF-PVEC|Li full batteries exhibited excellent rate capability (Figure 16g) and cycling performance (over 400 times) at a high current density of 1 C. These results demonstrated great potential of V-COF in SPEs.

**Figure 17:**
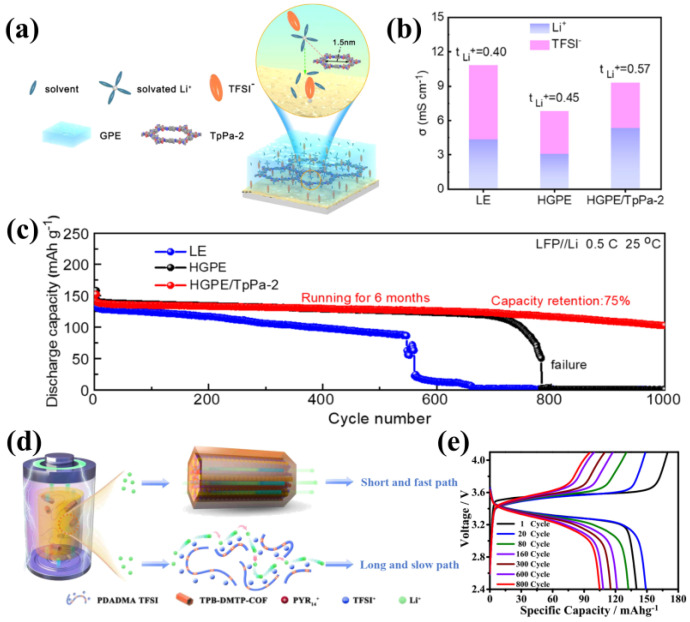
(**a**) Schematic illustration of desolvation behavior and structural transformation of SEI worked in HGPE/TpPa−2; (**b**) The ionic conductivity and Li^+^ transference number of LE, HGPE, and HGPE/TpPa−2; (**c**) Cycle performance of LiFePO_4_|HGPE/TpPa−2|Li full cell at 0.5 C. Reproduced with permission from Ref. [82], Copyright (2022), Elsevier.; (**d**) Schematic diagram of lithium−ion conduction path in IGE; (**e**) Charge and discharge curves of LiFePO4/IGE/Li cell. Reproduced with permission from Ref. [83], Copyright (2021), American Chemical Society.

COFs have the ability to regulate the solvation structure of lithium ions though their functional groups and pore structure. In a recent study, Chen and colleagues used COFs as fillers in a gel polymer electrolyte matrix to create batteries with exceptional cycling performance and safety [82]. Specifically, they introduced amino- and aldehyde-rich TpPa-2 to the gel polymer electrolyte via one-step radiation polymerization to promote desolvation of Li^+^ and modulate the composition of SEI (Figure 17a). This effectively inhibited the growth of lithium dendrites, resulting in improved safety and stability of the batteries. The addition of only 0.375 wt% of TpPa-2 increased the ionic conductivity from 6.8 mS/cm (in the pure gel electrolyte system) to 9.3 mS/cm, and broadened the electrochemical window to 4.8 V. The COFs also limited the anion movement, and the ion mobility number was increased from 0.45 to 0.57 (Figure 17b). The Li-Li symmetric bateries with the composite electrolyte were stable cycled at a current density of 0.5 mA/cm^−2^. Furthermore, the LFP|HGPE/TPPA-2|Li full cell exhibited superior cycling performance, with 75% capacity retention even after 1000 cycles (Figure 17c), fully validating the role of COFs when used as electrolyte fillers.

Besides solid polymer electrolytes (SPEs), ionic gel electrolytes (IGEs) comprising polymeric ionic liquids and ionic liquids can serve as matrix materials for COFs. The unique nanostructure of COFs enables effective accommodation of ionic liquids, allowing for high loadings of ionic liquids, while also providing fast ion channels that enhance ionic conductivity. In 2021, Sun’s team studied the influence of adding 2D COFs as fillers to ionic liquids and constructed a high-performance ionogel electrolytes (IGEs) [83] (Figure 17d). They prepared TB-DMTP-COF nanospheres with a diameter of 800 nm, and added them into PDADMA TFSI/PYR14TFSI (polymeric ionic liquid/ionic liquid, PIL/IL) matrix. Owing to the stronger binding ability of TPB-DMTP-COF to anions and its ordered nanochannels, when 3.2 wt% COFs were added into PIL/IL, the ionic conductivity reached 2.8 × 10^−4^ S cm^−1^ at 30 °C, which was 2.2 times of that without COFs, and the electrochemical window increased to 5 V. Assembled to LiFePO_4_||IGE||Li cells, the discharge capacity reached 107 mAh g^−1^ after 800 cycles at 1 C and 60 °C (Figure 17e). As a consequence, the higher transport behavior of ILs confined in the pore of the COFs was found compared to that in the PILs.

The major performance of SSEs with different COF fillers are summarized in Table 2.

As described above, COFs have great merits as fillers for SPEs or IGEs. The crystalline porous structure of COFs and the stable ordered channels in a wide temperature range significantly contribute to promoting ion transport. Moreover, the ease of functionalization of COFs allows for integration of various functional groups to achieve specific goals, achieving such as attaining high-voltage-tolerance, guiding uniform Li deposition. or regulating solvent structure. Generally, the introduction of COFs can enhance the mechanical properties of the polymer matrix and effectively inhibit the growth of lithium dendrites. However, it is a remarkable fact that there commonly exists a threshold effect when COFs are used as fillers. The excessive addition of COFs can negatively impact dispersion, leading to a decrease in ionic conductivity. The morphology and size of COFs will also affect the dispersion. Therefore, it is crucial to explore the appropriate addition of COFs with various morphology and size to achieve optimal electrochemical performance. In addition, the combination of the COF filler and the matrix, as well as the synergistic effect between the matrix and the filler on ion transport, should be thoroughly investigated in the subsequent research to prepare high-performance composite electrolytes.

## 5. Conclusions and Outlook

In this review, we summarized the advances of COFs in SSEs for lithium metal batteries over the past decade in order to analyze the relationship between the structure of COFs and the ion conduction mechanism. We also comprehensively sorted out the strategies utilized to achieve superior electrochemical performance of COFs in solid electrolytes, including their applications as host and as fillers. COFs have exhibited great application potential in SSEs due to their unique structural characteristics.

Despite the significant progress made in researching COFs as SSEs there remain a number of important scientific issues that require further investigation. Structural design, theoretical study, and practical applications are among the foremost areas of concern. Specifically, the long-range ordered nanochannels of COFs play a significant role in ionic conduction, but the relationships between structure and performance remains unclear. The ionic transport behavior in different types of COFs is also complicated, and host-guest interaction must be considered. To design high-performance SSEs, the ion transport mechanism must be elucidated. In addition, the concentration of mobile ions in the nanochannels of COFs is still low, which limits ionic conductivity and hinders practical application. Finally, the complicated preparation steps and immature processing technology of COFs must be overcome to realize their potential as SSEs in practical applications.

In light of the aforementioned issues, in order to comprehensively improve the electrochemical performance and practicality of COFs, future research should be directed to following areas:

(1) It is essential to systematically analyze the ion transport mechanisms in COFs in combination with advanced characterization techniques, such as 2D exchange NMR, NMR relaxometry, variable-temperature NMR, pulsed field gradient NMR and time of flight secondary ion mass spectrometry (TOF-SIMS), and computational simulation, such as molecular dynamics simulation and first-principles calculation). Furthermore, it is also crucial to establish the structure–property relationships of COFs, including the specific relationship between the size, morphology, pore size, interplanar spacing and specific surface area on ionic conduction. For functionalized COFs, the relationships between the type, density, spatial arrangement of functional groups and ion transport also need to be systematically studied, so as to achieve purposeful structural design.

(2) Solvents and plasticizers are commonly introduced into COFs for further enhanced ionic conductivity. Yet the addition of solvents not only reduces the Li^+^ transference number, but also brings high flammability, which violates the original intention of solid-state electrolytes to pursue safety. Therefore, future research could explore the preparation of solvent-free COFs derived solid electrolytes. In fact, different strategies for different types of COFs should be adopted according to their ionic conduction mechanism. For electroneutral COFs, the synergistic effects of different lithium salts, solvents and polymer chains should be considered. For cationic COFs, the influence of different functional groups should be analyzed. As for anionic COFs, it is vital to introduce groups with moderate delocalization ability, and it is also important to optimize the spatial distribution and density of hopping sites. When COFs are used as fillers, the pore wall decoration and the host-guest interaction should be considered adequately. In order to increase the concentration of charge-carriers in COFs, adding extra weakly coordinating lithium salts and decoupling Li^+^ cations by different methods can by also effective strategies. In addition, existing studies suggest that exfoliation into nanosheets, mechanical stress pressing, and heat treatment can affect orientation, and constructing anisotropic COFs is more beneficial to ionic conduction.

(3) To increase the industrial applicability of COFs, it is important to develop synthesis strategies that are more rapid, simple and low-cost COFs. The harsh sealed condition, high reaction temperature and relatively long reaction time of traditional solvothermal synthesis of COFs limit their practical use. Instead, various novel synthesis methods have emerged in recent years, such as hydrothermal synthesis [84], mechanochemical-assisted synthesis [85], microwave-assisted solvothermal synthesis [86], interfacial synthesis [87] have emerged and gradually achieved the rapid preparation of COFs. To cope with the poor processability of COFs, incorporate them with polymers to form flexible self-standing SSEs is one of the strategies. Future research should therefore focus on the preparation of COF-SSEs with high flexibility and mechanical strength.

In conclusion, the construction of high-performance ion-conducting COFs is still confronted with great challenges. The strategies discussed in this review provide potential solutions to overcome these obstacles. The continued study on ion-conducting COFs can potentially pave the way for the next generation high-performance all-solid-state batteries.

## Figures and Tables

**Table 1:** Summary of the major performances of COF-based SSEs.

Electrolyte							Li Metal Battery						Ref.
COFs	Li Salt	Solvent	σ (S cm^−1^)	t_Li_^+^	E_a_ (eV)	EW (V)	Cathode	Cycling Conditions		Q_dis1_ (mAh/g)	Capacity Retention		
											Cycle	%	
**ICOF-2**		PC	3.05 × 10^−5^ (RT)	0.80	0.24	4.5							[65]
**COF-5**	LiClO_4_		2.6 × 10^−4^ (RT)		0.37								[43]
**TpPa-1**	LiClO_4_		1.5 × 10^−4^ (RT)										[43]
**CD-COF**	LiPF_6_	EC/DMC	2.7 × 10^−3^ (30 °C)		0.26								[66]
**Li-CON-TFSI**	LiTFSI		5.74 × 10^−5^ (30 °C)		0.61	3.8							[60]
**TPB-DMTP-COF**	LiClO_4_		1.36 × 10^−7^ (40 °C)		0.96								[47]
**TPB-BMTP-COF**	LiClO_4_		6.04×10^−6^ (40 °C)		0.87								[47]
**PEG-Li^+^@COF-5**	LiClO_4_		3.60 × 10^−8^ (30 °C)	0.40	0.35								[48]
**PEG-Li^+^@COF-300**	LiClO_4_		1.40 × 10^−6^ (30 °C)	0.44	0.20								[48]
**PEG-Li^+^@EB-COF-ClO_4_**	LiClO_4_		1.93 × 10^−5^ (30 °C)	0.60	0.21					N/A			[48]
**PEG--Li^+^@CD-COF-Li**	LiClO_4_		2.60 × 10^−5^ (30 °C)	0.20	0.17								[48]
**TpPa-SO_3_Li**			2.7 × 10^−5^ (RT)	0.90	0.18	4.0							[74]
**H-Li-ImCOF**		PC	5.3 × 10^−3^ (RT)	0.88	0.12	4.5							[71]
**CH_3_-Li-ImCOF**		PC	8.0 × 10^−3^ (RT)	0.93	0.27	4.5							[71]
**CF_3_-Li-ImCOF**		PC	7.2 × 10^−3^ (RT)	0.81	0.10	4.5							[71]
**DBC-2P@PEG**	LiBF_4_		2.1 × 10^−3^ (70 °C)		0.09								[49]
**Ge-COF-1**			1.1 × 10^−6^ (30 °C)		0.26								[68]
**Ge-COF-1**	LiPF_6_	EC/DEC	2.5 × 10^−4^ (30 °C)	0.67	0.29								[68]
**COF-PEO-3-Li**	LiTFSI		9.73 × 10^−5^ (200 °C)										[50]
**COF-PEO-6-Li**	LiTFSI		3.71 × 10^−4^ (200 °C)										[50]
**COF-PEO-9-Li**	LiTFSI		1.33 × 10^−3^ (200 °C)			5.2	LiFePO_4_	2.5–4.2 V, 0.02 C (100 °C)		120	10	91.7	[50]
**[TEO]_0.33_TPB-DMTP-COF**	LiClO_4_		7.77 × 10^−6^ (40 °C)		0.78								[51]
**[TEO]_0.5_TPB-DMTP-COF**	LiClO_4_		1.31 × 10^−5^ (40 °C)		0.68								[51]
**[TEO]_0.33_TPB-BMTP-COF**	LiClO_4_		8.43 × 10^−6^ (40 °C)		0.87								[51]
**[TEO]_0.5_TPB-BMTP-COF**	LiClO_4_		5.51 × 10^−6^ (40 °C)		0.82								[51]
**[TEO]_1_TPB-BPTA-COF**	LiClO_4_		9.04 × 10^−7^ (40 °C)										[51]
**dCOF-ImTFSI-60@Li**	LiTFSI		9.74 × 10^−5^ (30 °C)	0.72	0.28	5.3	LiFePO_4_	2.5–4.0 V, 0.1 C (80 °C)		143.7	40	98.3	[63]
**Tp-PaSO_3_Li-COFs**		EC/DMC	1.6 × 10^−3^ (20 °C)	0.94	0.13		LiFePO_4_	2.5–3.6 V, 0.2 C		152	N/A	N/A	[75]
**Im-COF-TFSI@Li**	LiTFSI		2.92 × 10^−5^ (30 °C)	0.62	0.32	4.2	LiFePO_4_	2.5–4.0 V, 0.1 C (80 °C)		123.3	100	91.6	[64]
**Li-CON-1**			2.13 × 10^−7^ (20 °C)	0.86	0.25	4.2							[72]
**Li-CON-2**			4.36 × 10^−6^ (20 °C)	0.83	0.22	3.8							[72]
**Li-CON-3**			3.21 × 10^−5^ (20 °C)	0.92	0.13	4.3	BQ	1.7–4.0 V, 1 C (20 °C)		214.8	500	87.7	[72]
**LPC-2**	LiI	P1,1I	8.9 × 10^−4^ (40 °C)	0.58	0.23		LiFePO_4_	2.5–3.6 V, 0.2 C		144.6	35	68.6	[53]
**ACOF**	LiPF_6_	EC/DEC	3.7 × 10^−3^ (20 °C)	0.82	0.15		LiCoO_2_	3.0–4.5 V, 0.2 C (25 °C)		180	500	70	[69]
**COF-NUST-7**	LiTFSI	PyR_14_TFSI	9.66 × 10^−4^ (120 °C)	0.11	0.317	4.2	LiFePO_4_	2.5–3.8 V, 0.05 C (100 °C)		140.8	60	93.3	[54]
**COF-NUST-8**	LiTFSI	PyR_14_TFSI	1.4 × 10^−3^ (120 °C)		0.301								[54]
**COF-NUST-9**	LiTFSI	PyR_14_TFSI	2.3 × 10^−3^ (120 °C)		0.323								[54]
**TAPB-TPA**	LiTFSI	PyR_14_TFSI	8.7 × 10^−4^ (30 °C)	0.29			LiFePO_4_	2.5–4 V, 0.1 C (60 °C)		163.1	50	92.2	[55]
**Q-COF**	LiTFSI		7.5 × 10^−5^ (30 °C)	0.72	0.15	5.6	NMC811	3.0–4.4 V (60 °C)		160	400	82	[70]
**PEG-Li^+^@COF-M**	LiTFSI		2.2 × 10^−5^ (20 °C)		0.46	5.0							[48]
**DMA@COF-SO_3_Li**	LiTFSI		1.65 × 10^−4^ (23 °C)	0.85		4.5	LiFePO_4_	2.8–3.8 V, 0.5 C (65 °C)		122	130	90.2	[76]
**LiOOC-COF1**			7.23 × 10^−7^ (30 °C)	0.82	0.21	3.5							[73]
**LiOOC-COF2**			2.66 × 10^−6^ (30 °C)	0.86	0.2	4.06							[73]
**LiOOC-COF3**	LiPF_6_	EC/DEC	1.36 × 10^−5^ (30 °C)	0.91	0.17	4.2	C_6_O_6_	1.5–4 V, 50 mA g^−1^ (25 °C)		418	500	89.7	[73]

Note: σ, tLi^+^, Ea, EW, Qdis1 trepresent the ionic conductivity, Li^+^ transference number, activation energy and electrochemical window of different COF-based SSEs and initial discharge capacity of Li metal battery.

**Table 2:** Summary of the major performances of SSEs with COF filler.

Electrolyte								Li Metal Battery					Refs.
The Composition of Electrolyte				Filler Content (wt%)	σ (S cm^−1^)	t_Li_^+^	EW (V)	Cathode	Cycling Conditions	Q_dis1_ (mAh/g)	Capacity Retention		
Matrix	COFs	Li Salt	Solvent								Cycle	%	
PVDF	H-COF-1@10	LiClO_4_		20	2.74 × 10^−4^ (RT)	0.71	4.3	LiFePO_4_	1 C (RT)	128	80	94	[78]
PEO	ivCOF-2-Br	LiTFSI		10	3.84 × 10^−4^ (20 °C)								[79]
PDADMA TFSI	TPB-DMTP-COF	LiTFSI	PYR14TFSI	3.2	2.8 × 10^−4^ (30 °C)	0.16	5 V	LiFePO_4_	1 C (60 °C)	140	800	76.4	[83]
PDM (PEGDA/ PEGMA)	V-COF	LiTFSI		1	1.1 × 10^−4^ (40 °C)	0.45	5.0	LiFePO_4_	1 C (40 °C)	136	300	83.8	[80]
PVEC	V-COF	LiTFSI		2	1.1 × 10^−4^ (40 °C)			LiFePO_4_	1 C (40 °C)	125	400	82.25	[81]
AN+PEGDMA	TpPa-2	LiTFSI	EC/DEC	0.375	9.3 × 10^−3^ (RT)	0.57	4.8	LiFePO_4_	0.5 C (RT)	~140	850	85	[82]

Note: σ, tLi^+^, Ea, EW, Qdis1 trepresent the ionic conductivity, Li^+^ transference number, activation energy and electrochemical window of different COF-based SSEs and initial discharge capacity of Li metal battery.

## Data Availability

The data presented in this study are available upon request from the corresponding author.
